# Insertion of a chimeric retrotransposon sequence in mouse *Axin1* locus causes metastable kinky tail phenotype

**DOI:** 10.1186/s13100-019-0162-7

**Published:** 2019-05-03

**Authors:** Zhuqing Wang, Hayden McSwiggin, Simon J. Newkirk, Yue Wang, Daniel Oliver, Chong Tang, Sandy Lee, Shawn Wang, Shuiqiao Yuan, Huili Zheng, Ping Ye, Wenfeng An, Wei Yan

**Affiliations:** 1 Department of Physiology and Cell Biology, University of Nevada School of Medicine Center for Molecular Medicine, Room 207B 1664 North Virginia Street MS/0575, Reno, NV 89557 USA; 2 Avera McKennan Hospital and University Health Center, Sioux Falls, SD 57108 USA; 3 Department of Pharmaceutical Sciences, South Dakota State University, Brookings, SD 57007 USA; 4 Department of Obstetrics and Gynecology, University of Nevada, Reno School of Medicine, Reno, NV 89557 USA; 5 Department of Biology, University of Nevada, Reno, Reno, NV 89557 USA

**Keywords:** Retrotransposon, CRISPR/Cas9, LINE-1, IAP, MaLR, Alternative splicing, Histone modification, DNA methylation, Epigenetic inheritance

## Abstract

**Background:**

Transposable elements (TEs) make up > 50% of the human genome, and the majority of retrotransposon insertions are truncated and many are located in introns. However, the effects of retrotransposition on the host genes remain incompletely known.

**Results:**

We report here that insertion of a chimeric L1 (cL1), but not IAP solo LTR, into intron 6 of *Axin1* using CRIPSR/Cas9 induced the kinky tail phenotype with ~ 80% penetrance in heterozygous *Axin*^*cL1*^ mice. Both penetrant (with kinky tails) and silent (without kinky tails) *Axin*^*cL1*^ mice, regardless of sex, could transmit the phenotype to subsequent generations with similar penetrance (~ 80%). Further analyses revealed that a longer *Axin1* transcript isoform containing partial cL1-targeted intron was present in penetrant, but absent in silent and wild type mice, and the production of this unique *Axin1* transcript appeared to correlate with altered levels of an activating histone modification, H3K9ac.

**Conclusions:**

The mechanism for *Axin*^*cL1*^ mice is different from those previously identified in mice with spontaneous retrotransposition of IAP, e.g., *Axin*^*Fu*^ and *A*^*vy*^, both of which have been associated with DNA methylation changes. Our data suggest that *Axin1* locus is sensitive to genetic and epigenetic alteration by retrotransposons and thus, ideally suited for studying the effects of new retrotransposition events on target gene function in mice.

**Electronic supplementary material:**

The online version of this article (10.1186/s13100-019-0162-7) contains supplementary material, which is available to authorized users.

## Background

Transposable elements (TEs) make up > 50% of the human genome [1]. The vast majority of human TEs are retrotransposons, which replicate via a RNA-based process termed retrotransposition [2]. Based on sequence organization, retrotransposons are further classified into LTR (long terminal repeat) and non-LTR retrotransposons. LTR retrotransposons are also called endogenous retroviruses (ERVs), which display ongoing insertional activities in mice but not in humans [3]. Among them, mammalian apparent LTR retrotransposon (MaLR) elements are the most abundant in both human and mouse genomes although they are no longer replicating [3–6]. On the other hand, IAP (intracisternal A-type particle) is one of a few LTR retrotransposon families that remain active in the mouse genome [3–6]. Non-LTR retrotransposons include long interspersed elements (LINEs) and short interspersed elements (SINEs). LINE-1 (L1) sequences are abundant (~ 17% of the human genome) and have been identified as the only active and autonomous mobile element in the human genome [2, 3]. L1 consists of four components: a 5′ untranslated region (UTR) that serves as a promoter, a 3′ UTR containing a polyadenylation signal, an ORF1 (open reading frame 1) encoding an RNA binding protein with nucleic acid chaperone activity, and a conserved ORF2 protein that harbors reverse transcriptase and endonuclease activities [2]. In addition to self-mobilization, L1 proteins can also copy other RNAs into a new locus via several distinct pathways. SINEs, such as human Alu and SVA (SINE-VNTR-Alu) elements, hijack the L1 retrotransposition machinery and have successfully proliferated in the human genome [7–9]. Although not as efficient, non-TE transcripts can also be copied, forming processed pseudogenes [10, 11]. The sequence downstream to a full-length L1 can be mobilized to new locations via 3′ transduction [12–15]. Indeed, a study of 244 cancer patients has revealed that almost 25% of patients have 3′ transductions of L1 sequence [16]. Chimeric or hybrid sequences can be generated when L1 reverse transcriptase switches templates [17–19]. The vast majority of TEs in the genome are truncated or rearranged, leaving behind 3′ fragments of L1 s or single (“solo”) LTRs of ERVs, in which ORFs critical for TE replication are lacking [2, 3, 20, 21]. Moreover, most of the TE insertions described to date in cancers are intronic or intergenic [22, 23]. It remains to be investigated the extent to which TE insertions affect the expression of their host coding genes and genomic activities near the insertion sites.

It is difficult to study retrotransposition and its effects on gene expression because retrotransposon sequences are widespread in the genome and often integral parts of the introns of coding genes [22]. One approach is to follow the fate of de novo insertions that are launched from engineered donor L1 transgenes. In this regard, several cell and mouse models have been generated to study the effects of L1 retrotransposition by tagging human or mouse L1 s with intron-disrupted retrotransposition reporters [24–29]. This approach has indeed provided important insights into L1 retrotransposition activities in various cell lines and tissues [26–31]. In several cell types (e.g. mouse embryonic stem cells, rat neuronal progenitor cells, human embryonic carcinoma and other cancer derived cell lines), the newly integrated L1 s are efficiently silenced by epigenetic marks, such as DNA methylation, histone deacetylation or H3K9me3 (H3 Lys9 trimethylation) [26–29]. In mouse models, when propagated through the germline, the retrotransposed sequences exert a graded influence on the flanking genomic sequences at the level of DNA methylation, creating “sloping shores” around the hypomethylated CpG island in germ cells [32]. A limitation of this approach is that both the site and the length of insertions are unpredictable. So it is impossible to compare the effect of different retrotransposon sequences on flanking genes. A complementary approach is to study the effect of spontaneous insertional mutagenesis by endogenous retrotransposition events in mice [33] and in humans [34]. However, these insertions are fixed, some insertions of retrotransposons in these loci may have no discernable phenotype, and therefore, the effects of these insertions remain to be elucidated. We sought to find some DNA loci that could result in discernable phenotypes to study the effects of L1 retrotransposition. Interestingly, two of most studied mouse models involve spontaneous LTR retrotransposon (e.g. IAP) insertions. The first is *Axin*^*Fu*^*(Axin*^*Fused*^*)* mice, in which a 5.1-kb IAP retrotransposon is inserted in antisense orientation into intron 6 of *Axin1*, causing a kinky tail phenotype [35]. The second case is *A*^*vy*^ (agouti viable yellow) mice, which show variable yellow agouti coat color phenotypes and is, similar to *Axin*^*Fu*^, caused by a 5.1-kb IAP insertion in antisense orientation into the pseudoexon 1A of *agouti* (*A*) locus [36]. In both cases, DNA methylation levels of the IAP retrotransposon appear to inversely correlate with the severity of the phenotype. Additionally, both the DNA methylation patterns and phenotypes can be transmitted to subsequent generations in a metastable manner [37–40]. To study the effects of retrotransposed sequences, we attempted to generate mutant mice carrying either a shorter version of IAP LTR (e.g., a solo LTR) or a chimeric L1 (cL1) at the same sites in *Axin1* and *A (agouti)* loci using the CRISPR/Cas9 technology [41–43]. Surprisingly, we failed to recapitulate the phenotypes when the solo LTR of IAP was inserted into the same two loci as those in the *Axin*^*Fu*^ and *A*^*vy*^ mice. Of interest, we did observe kinky tail phenotype when the cL1 was inserted into intron 6 of *Axin1* (termed *Axin*^*cL1*^). Moreover, we found that the molecular mechanisms underlying the kinky tail phenotype were different between *Axin*^*cL1*^ and *Axin*^*Fu*^ mice.

## Results

### Insertion of a chimeric L1, not the IAP solo LTR, into intron 6 of *Axin1* induced the kinky tail phenotype

To test whether an IAP solo LTR can induce the kinky tail phenotype, we first inserted a 335 bp IAP solo LTR flanked by two loxP sites in reverse orientation into intron 6 of *Axin1* using CRISPR/Cas9 (Additional file 1: Figure S1A and supplemental notes). The insert only contains the LTR of IAP identified in *Axin*^*Fu*^ mice and has been shown to function as a cryptic promoter in those mice [38]. One founder was obtained, but with no kinky tail phenotype although both PCR-based genotyping and Sanger sequencing results showed that the IAP solo LTR was indeed inserted precisely (Additional file 1: Figure S1A). In *Axin*^*Fu*^ mice, not all displayed the kinky tail phenotype; some have normal tails because of hypermethylated IAP [38]. When those silent *Axin*^*Fu*^ mice are bred with wild type (WT) mice, a small proportion of their offspring do display the kinky tail phenotype [38]. Thus, a lack of the kinky tail phenotype in the founder obtained could be due to either that the IAP solo LTR alone could not induce the kinky tail phenotype, or that the insert got silenced in founder mice. To test the two possibilities, we crossed the *Axin*^*IAP*^ founder with WT mice, but none of > 20 *Axin1*^*+/IAP*^ F1 mice showed the kinky tail phenotype, suggesting that the IAP solo LTR insertion does not disrupt *Axin1* gene expression and thus, induces no kinky tail phenotype. Similarly, no variable yellow agouti coat color phenotype was found in either of the founder (F0) or 21 F1 mice when an antisense IAP solo LTR, which is the same as that identified in *A*^*vy*^ mice [37, 39, 40], was inserted into the *A* (agouti) locus (Additional file 1: Figure S1B and supplemental notes).

**Fig. 1:**
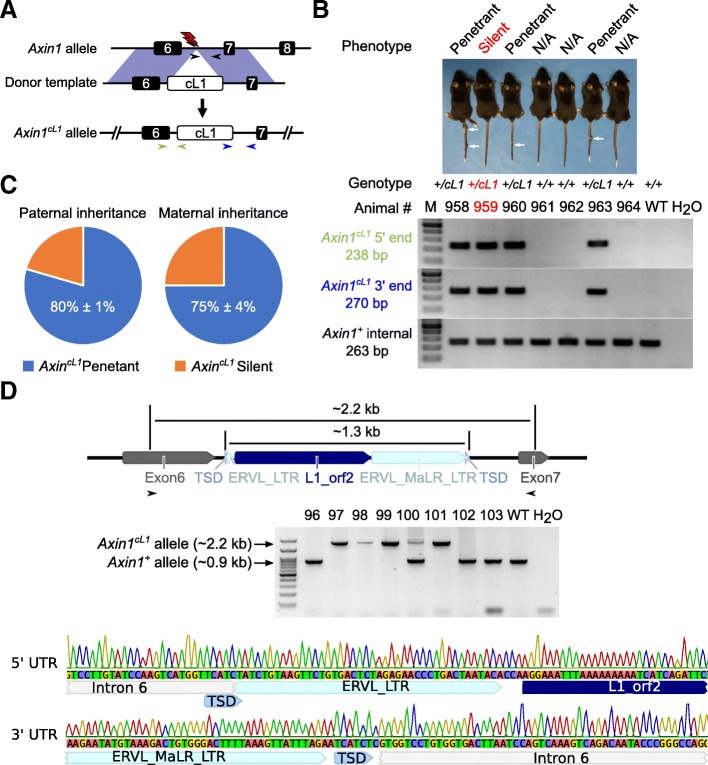
Generation of *Axin1*^*cL1*^ mice using CRISPR/Cas9 and phenotypic characterization. a Schematics showing the strategy for generating *Axin*^*cL1*^ mice. The red lightning bolt represents the gRNAs used to target the reverse strands of the genomic DNA. The black arrows show the position of internal primers in the *Axin1*^*+*^ allele, whereas the blue and the light green arrows indicate those for amplifying the 5′ and 3’ends of the *Axin1*^*cL1*^ allele, respectively. The expected size of PCR products is indicated in the same color in the lower panels of **b**. **b** Image of a representative litter of seven F5 mice derived from a F4 silent *Axin*^*cL1*^*(Axin1*^*+/cL1*^) female mouse bred with a WT male, including three penetrant (white arrows pointing to the kinked regions in the tails), one silent *Axin*^*cL1*^ mouse and three WT (*Axin1*^*+/+*^) littermates (Upper panel). Genotyping results of these mice are shown in lower panels and the positions of the three sets of primers used are marked in **a**. **c** Pie charts showing the distribution of penetrant (with kinky tails) vs. silent (without kinky tails) *Axin*^*cL1*^ (*Axin1*^*+/cL1*^) mice in an outbreeding scheme (*Axin1*^*+/cL1*^ × WT) across three generations. Data are represented as means ± SEM (*n* = 353 for paternal transgenerational inheritance, and *n* = 425 for maternal transgenerational inheritance). **d** Confirmation of cL1 insertion into intron 6 of *Axin1*. Long-range PCR was used to amplify fragments derived from *Axin*^*cL1*^ (~ 2.2 kb) and WT (~ 0.9 kb) alleles (upper and middle panels), and the PCR products were sequenced to confirm the successful cL1 insertion in intron 6 of *Axin1*(lower panels). TSD, target site duplications; ERVL_LTR, LTR of MT2_Mm in ERVL family (5′ extra nucleotide); L1_orf2, orf2 of Lx2 in L1 family; ERVL_MaLR_LTR, LTR of MT_int in ERVL_MaLR family, serving as the 5′ extra nucleotides

Next, we tested whether insertion of other repetitive sequences can induce the kinky tail phenotype. We generated a repetitive sequence, called chimeric L1 (cL1) herein, consisting of a partial Orf2 of L1 and an LTR of MaLR, and inserted it into intron 6 of *Axin1* using the CRISPR/Cas9 (Fig. 1 a and d and Additional file 1: supplemental notes). To represent a retrotransposed sequence, we also included 6 bp target site duplications (TSDs) and 44 bp 5′ extra nucleotides in the cL1 donor construct (Fig. 1d and Additional file 1: supplemental notes). We chose to use this specific chimeric L1 to mimic retrotransposition in vivo for the following reasons: First, such a chimeric sequence may result from template switching or transduction during retrotransposition, which is a pervasive phenomenon in both human and mouse genomes [17–19]. Second, the 762 bp Orf2 of L1 (Fig. 1d and Additional file 1: supplemental notes), which harbors a partial Z-motif and a partial reverse transcriptase domain, is highly conserved among different L1 families [44–46]. When aligning the Orf2 sequence against the mm10 genome with BLAT [47], one exact match was found on chromosome 3:76892981–76,893,742 and 70 other hits showed > 80% sequence identity (Additional file 2: Table S1). Moreover, the MaLR elements are the most abundant LTR retrotransposon sequences in both human and mouse genomes [3]. When aligning the LTR of the MaLR to the mouse genome mm10, 20 perfect matches were found and over 200 other hits showed > 96% sequence identity (Additional file 3: Table S2). Therefore, insertion of the two conserved regions (L1 Orf2 and MaLR LTR) of TEs into the genome allows for studying the combined genetic/epigenetic impact at this locus. Finally, both TE fragments have no retrotransposition capability, which is further confirmed by our assays for DNA copy number variation (Additional file 1: Figure S1C).

We obtained 3 founders (F0) carrying the cL1 insertion, which was confirmed by Sanger sequencing of the long-range PCR products containing the full-length cL1 (Fig. 1d). One of the three founders showed a strong kinky tail phenotype, while the other two had normal tails, despite the same genotype (*Axin1*^*+/cL1*^). By further breeding the F0 s with WT mice, we obtained F1 heterozygous mice. Intercrossing F1 heterozygous mice produced WT, heterozygous and homozygous F2 mice at the Mendelian ratio (Fig. 1d). All homozygous (*Axin1*^*cL1/cL1*^) mice showed kinky tails and also displayed neuronal abnormalities characterized by motor discordances (e.g., spinning with shaky heads and imbalance), whereas ~ 80% of the heterozygous (*Axin1*^*+/cL1*^) mice showed the kinky tail phenotype and the remaining heterozygous mice had normal tails (Fig. 1 b and c). These results suggest that a chimeric L1/MaLR sequence, rather than IAP solo LTR, can cause the kinky tail phenotype once inserted into intron 6 of *Axin1* in mice.

### Stable transmission of the kinky tail phenotype with a fixed penetrance across multiple generations

**Fig. 2:**
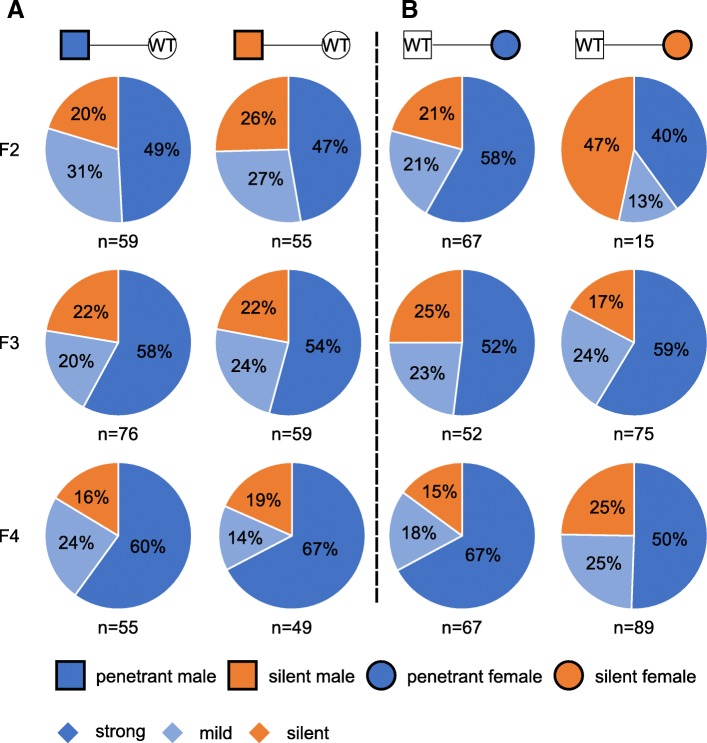
Transgenerational inheritance of the kinky tail phenotype in *Axin*^*cL1*^ (*Axin1*^*+/cL1*^) mice. **a** Paternal transgenerational inheritance of the kinky tail phenotype among *Axin*^*cL1*^ mice. Male penetrant (left panel) and silent (right panel) *Axin*^*cL1*^ mice were bred with wild type (WT) females, and the percentage of strong (dark blue) or mild (light blue) kinky and silent (orange) *Axin*^*cL1*^ offspring, as well as the number of mice counted are indicated (Note that WT pups were excluded from the analyses). **b** Maternal transgenerational inheritance of the kinky tail phenotype among *Axin*^*cL1*^ mice. Female penetrant (left panel) and silent (right panel) *Axin*^*cL1*^ mice were bred WT females, and the percentage of strong (dark blue) or mild (light blue) kinky and silent (orange) *Axin*^*cL1*^ offspring, as well as the number of mice counted are indicated (Note that WT pups were excluded from the analyses)

*Axin*^*Fu*^ (*Axin1*^*+/Fu*^) mice showed kinky tails with highly variable severity, and the penetrant *Axin*^*Fu*^ mice (with strong or mild kinky tails) produced more penetrant offspring compared to those silent ones (without kinky tails) [38]. This phenotype can be transmitted to the next generation in a metastable manner, and the phenotypic variability correlates with the methylation status of the IAP retrotransposed into intron 6 in the offspring [38]. To examine whether the kinky tail phenotype induced by cL1 also displays a similar variability in phenotypic severity, we conducted breeding experiments to test the transmission of the phenotype through either paternal or maternal germline across three generations. Heterozygous *Axin*^*cL1*^ (*Axin1*^*+/cL1*^) penetrant and silent male F2 s were bred with WT females, and ~ 80% of the *Axin*^*cL1*^ offspring (F3 s) were penetrant mice (Fig. 2a). Further breeding of the penetrant and silent F3 and F4 *Axin*^*cL1*^ males and females with WT controls led to F4 and F5 *Axin*^*cL1*^ offspring displaying the kinky tail phenotype with similar penetrance (~ 80%) (Fig. 2a). The phenotypic penetrance stayed the same (at ~ 80%) across all three generations when the cL1 insertion was propagated through either the paternal or the maternal germline (Fig. 1c and Fig. 2). Among the penetrant *Axin*^*cL1*^ mice, the ones with stronger kinky tails accounted for ~ 70% across all three generations, regardless of paternal or maternal inheritance (Fig. 2a and b), suggesting the kinky tail phenotype can be stably inherited transgenerationally as long as the cL1 insertion exists.

### The kinky tail phenotype in *Axin*^*cL1*^ mice is caused by an aberrantly spliced *Axin1* transcript

**Fig. 3:**
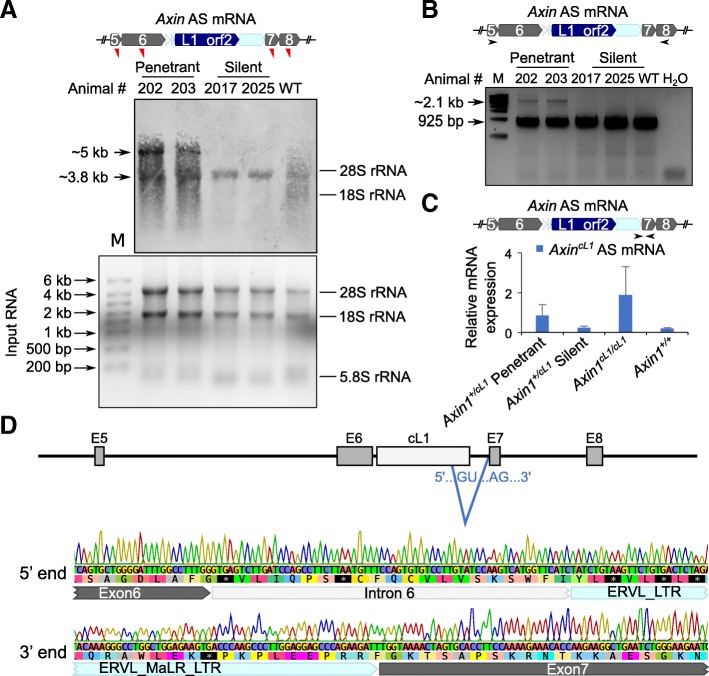
A longer *Axin1* transcript isoform containing partial cL1 is exclusively expressed in the penetrant *Axin*^*cL1*^ mice. **a** A representative Northern blot showing that the wild type transcript (~ 3.8 kb) was detected in *Axin*^*cL1*^ mice, both penetrant and silent, as well as wild type mice, whereas a longer transcript (~ 5 kb) was present only in penetrant *Axin*^*cL1*^ mice (middle panel). Red triangles indicate the relative positions of the probes used in the upper panel, and the total RNA inputs are shown in the lower panel. **b** RT-PCR detection of the longer transcript isoform unique to penetrant *Axin*^*cL1*^ mice. A pair of primers encompassing exons 5 and 8 (arrows in the upper panel) was used for PCR detection of WT (925 bp) and cL1-containing (~ 2.1 kb) transcripts (lower panel). **c** qPCR quantification of the longer transcript isoform unique to penetrant *Axin*^*cL1*^ and homozygous *Axin1*^*cL1/cL1*^ mice. Data are presented as means ± SEM, *n* = 3. **d** Schematic illustration of the alternative splicing event leading to the production of a longer transcript isoform unique to penetrant *Axin*^*cL1*^ mice (upper panel), as supported by the Sanger sequencing results (lower panel)

Retrotransposons inserted into the genome have been shown to act as a cryptic promoter, a terminator, and/or to induce alternative splicing [4, 29, 38, 48–53]. First, we performed dual luciferase reporter assays to test whether cL1 could function as a promoter (Additional file 1: Figure S2). Different parts of cL1 were amplified and used to replace the SV40 early promoter of *Renilla* luciferase reporter (*Rluc*) in the psiCHECK-2 vector. Promoter activity was detected in the antisense MaLR solo LTR, but not in other fragments, including the cL1 that we used to generate *Axin*^*cL1*^ mice (Additional file 1: Figure S2), indicating that cL1 does not function as a cryptic promoter in the context of the reporter construct. To test whether aberrant transcripts are produced, we performed Northern blot analyses with probes specific to *Axin1* exons 5, 6, 7 and 8 (Fig. 3a). Indeed, we found that a longer transcript was detected exclusively in penetrant mice (Fig. 3a). Consistent with Northern blot results, our RT-PCR analyses using primers specific to exons 5 and 8 also found an alternative splicing event in penetrant, but not in silent *Axin*^*cL1*^ mice (Fig. 3b). Sanger sequencing of the longer isoform revealed that part of the cL1 (L1-MaLR) sequence was included in the aberrant *Axin1* transcript, which was spliced at the canonical GU-AG splicing site, in penetrant mice (Fig. 3d and Additional file 1: supplemental notes). We further designed specific primers for the alternatively spliced *Axin1* transcript. qPCR confirmed that the alternative spliced *Axin1* transcript is exclusively expressed in penetrant *Axin*^*cL1*^ mice (Fig. 3c). Taken together, these data strongly suggest that the kinky tail phenotype in the *Axin*^*cL1*^ mice is induced by an aberrantly spliced *Axin1* transcript due to intron retention of cL1.

### Altered H3K9ac modification, rather than DNA methylation changes, correlates with the aberrantly spliced *Axin1* mRNA

**Fig. 4:**
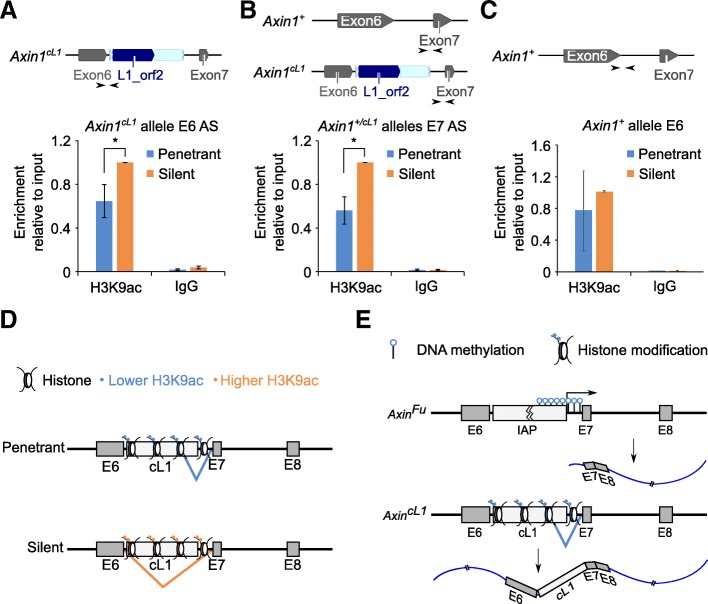
Reduced H3K9ac levels at the cL1 insertion site in penetrant *Axin*^*cL1*^ mice. **a** ChIP-qPCR analyses of H3K9ac levels using primers specific to the exon 6 splicing site of the *Axin1*^*cL1*^ allele. Arrows indicate relative locations of the primers used for ChIP-qPCR analyses (upper panel). Data are presented as means ± SEM, *n* = 3, *p < 0.05. **b** ChIP-qPCR analyses of H3K9ac levels using primers specific to the exon 7 splicing site of the *Axin1*^*cL1*^ and *Axin1*^*+*^ alleles. Arrows indicate relative locations of the primers used for ChIP-qPCR analyses (upper panel). Data are presented as means ± SEM, *n* = 3, *p < 0.05. **c** ChIP-qPCR analyses of H3K9ac levels using primers specific to the exon 6 splicing site of the *Axin1*^*+*^ allele. Arrows indicate relative locations of the primers used for ChIP-qPCR analyses (upper panel). Data are presented as means ± SEM, *n* = 3. **d** Schematic illustration showing the effect of reduced H3K9ac levels on splicing. Briefly, higher H3K9ac levels ensure correct splicing, which excludes cL1 from the transcript, whereas with lower H3K9ac levels, the cL1 tends to be retained and included in the transcript. **e** Comparison of the molecular mechanisms underlying the kinky tail phenotype between *Axin*^*Fu*^ and *Axin*^*cL1*^ mice. The kinky tail phenotype in *Axin*^*Fu*^ mice results from a shorter transcript isoform initiated from intron 6, and the phenotypic severity is inversely correlated with DNA methylation status, whereas the kinky tail phenotype in *Axin*^*cL1*^ mice is caused by a longer transcript isoform with cL1 intron retention, and the penetrance of the phenotype is fixed at 70–80%, and inversely correlated with H3K9ac levels at the cL1 insertion site

Despite the same genotype (*Axin1*^*+/cL1*^), only ~ 80% of *Axin*^*cL1*^ mice express the aberrant transcripts with partial cL1 retention. Therefore, epigenetic mechanisms are likely involved. Given that DNA methylation and histone modifications of the IAP LTR sequences in *Axin*^*Fu*^ mice have been correlated with the variable phenotypic severity [38, 54], we first examined DNA methylation of cL1 and its flanking regions in both penetrant and silent *Axin*^*cL1*^ mice. Surprisingly, bisulfite sequencing showed that DNA methylation patterns were not significantly altered between penetrant and silent *Axin*^*cL1*^ mice (Additional file 1: Figure S3 A and B). Additionally, no major changes in DNA methylation were found between the two groups by both methylated DNA immunoprecipitation (MeDIP) of 5-methylcytosine (5mC) and HhaI restriction enzyme (RE) digestion, which cleaves unmethylated GCGC site specifically, followed by qPCR (MeDIP-qPCR and RE-qPCR) (Additional file 1: Figure S3C). Taken together, these results suggest that the aberrant alternative splicing of *Axin1* transcript is not due to altered DNA methylation. Given that histone modifications (e.g. H3K9ac) affect alternative splicing [55–57], and H3K9ac and H4K20me3 marks have been associated with proper splicing of intron 6 of *Axin1* [54], we performed chromatin immunoprecipitation followed by qPCR (ChIP-qPCR) to examine H3K9ac levels (Fig. 4 a-c) at the *Axin*^*cL1*^ locus. Levels of H3K9ac, a histone mark for open chromatin structure, were much higher at the cL1 insertion site in the silent than in the penetrant mice (Fig. 4 a and b). These data suggest that a reduction in H3K9ac levels on the cL1 insertion site and its neighboring regions may affect splicing, leading to the production of a longer transcript containing cL1 (Fig. 4 d).

## Discussion

Mutant mice with variable yellow agouti coat color and kinky tail phenotypes were first reported 82 and 57 years ago, respectively [58, 59]. It was not until ~ 20 years ago that these phenotypes were correlated with spontaneous retrotransposition of IAPs in the mouse *A(agouti)* and *Axin1* loci, respectively [35, 36]. However, validation by inserting the IAP into these loci to recapitulate the phenotypes in different strains of mice has not been reported. Moreover, identification of a locus that is sensitive to retrotransposition, and tends to produce a visually discernable phenotype (e.g., kinky tails, coat color changes, etc.) as a result of functional disruptions would be ideal for investigating the effects of retrotransposition in vivo. To this end, we generated a number of mouse lines by inserting various repetitive sequences into exactly the same genomic location in either *A(agouti)* or *Axin1* locus as that reported in *A*^*vy*^ or *Axin*^*Fu*^ mice [37–40]. Interestingly, we found that insertion of IAP solo LTR induced no phenotypes, whereas insertion of a composite cL1 sequence into *Axin1* locus caused the kinky tail phenotype, which can be transmitted faithfully across multiple generations. These findings indicate that intronic retrotransposition events do not necessarily cause disruptions in the host genes leading to discernable phenotypes and that the effects of retrotransposition depend on sequence context and organization. Indeed, previous studies have shown that heterozygotes of 3 spontaneous mutations in *Axin1* gene, including *Axin*^*Fu*^ (*Axin*^*Fused*^), *Axin*^*Ki*^ (*Axin*^*Kinky*^*)* and *Axin*^*Kb*^ (*Axin*^*Knobby*^*),* all display the kinked tail phenotype, yet heterozygotes of a transgenic line called *Axin*^*Tg1*^ showed no phenotype [35]. Furthermore, *Axin*^*Fu*^ homozygotes are viable, whereas *Axin*^*Ki*^, *Axin*^*Kb*^ and *Axin*^*Tg1*^ homozygotes die around embryonic days 8–10 [35]. It is highly likely that the variable phenotypes among these strains reflect the positional effects of different insertions, e.g., the *Axin*^*Tg1*^ mice contain a ~ 600 bp transgene replacing exon 2, whereas *Axin*^*Fu*^ and *Axin*^*Kb*^ contain an IAP insertion in intron 6 and exon 7, respectively [35]. The lack of phenotype in mice carrying an insertion of IAP solo LTR into intron 6 of *Axin1* or pseudo exon 1A of *agouti (A)* loci is consistent with a recent report [21] showing that IAP LTR rarely displays promoter activity in vivo. Given that the IAP solo LTR sequence used was a part of the full-length IAP identified in *A*^*vy*^ and *Axin*^*Fu*^ mice, the negative finding hints that other parts of the full-length IAP sequences may contain certain hidden features (e.g., subtle sequence variations and/or RNA modifications), which are required for functional disruption of the host genes and consequently the induction of the kinky tail or variable yellow coat color phenotypes.

Although insertions of full-length IAP or cL1 into intron 6 of *Axin1* locus all induced the kinky tail phenotype, the underlying mechanisms appear to be different. In *Axin*^*Fu*^, IAP insertion into intron 6 compromises *Axin1* gene expression by producing a truncated transcript, which is inversely correlated to DNA methylation status [38]. In contrast, in our *Axin*^*cL1*^ mice, while the inserted cL1 sequence displays neither promoter activities in vitro nor DNA methylation changes in vivo, production of the aberrant transcript resulting from the retention of cL1 appears to correlate with significantly reduced levels of H3K9ac. Supporting our findings, reduced H3K9ac has been shown to cause alternative exon retention in *Ncam* (Neural cell adhesion molecule) due to decreased RNA polymerase processivity [56, 57]. Moreover, H3K9ac is also significantly more enriched in the IAP LTR of the *Axin*^*Fu*^ locus in embryos sired by penetrant males than those by silent males [54]. A recent study [53] reports that MaLR LTRs function as splicing donors rather than splicing acceptors, which is consistent with our data showing that the MaLR LTR in the cL1 serves as a splicing donor. While associations between H3K9ac levels and aberrant splicing have been established [55–57], the underlying mechanism remains elusive. In *Axin1*^*cL1*^ mice, the longer splicing variant containing partial chimeric L1 sequence possesses several premature termination codons (PTCs), which are well known to cause transcript degradation via the nonsense mRNA decay (NMD) pathway [60–62]. However, our Northern blot results revealed that the longer transcript, which is unique to the penetrant *Axin*^*cL1*^ mice, was nearly as abundant as the shorter wild-type one, suggesting that the splicing variant does not undergo NMD-mediated degradation. Therefore, it is highly likely that the longer splicing variant is translated into a mutant form of AXIN1 with a truncated C terminus lacking DIX domain, as compared to wild-type AXIN1. Unfortunately, we have not been able to identify a commercial antibody that could detect wild-type AXIN1 correctly (~92kD protein). Production of good AXIN1 antibodies and generation of a mouse model over-expressing the splicing variant/mutant AXIN1 lacking DIX domain would provide the ultimate evidence supporting the cause-effect relationship between the splicing variant/mutant AXIN1 without DIX domain and the kinky tail phenotype in the future. Together, our data suggest that the Axin1 locus is sensitive to genetic and epigenetic alterations caused by retrotransposition and thus, can serve as an ideal genomic location for studying the effects of retrotransposition on host gene expression and activities of nearby genome. With advancement of the CRISPR/Cas9 technology, TEs of interest can easily be inserted into the *Axin1* locus and the effects of various TEs on *Axin1* and nearby genome can be analyzed in vivo.

Transgenerational epigenetic inheritance of the variable yellow agouti coat color and kinky tail phenotypes in *A*^*vy*^ and *Axin*^*Fu*^ mice is of great interest although the underlying mechanism remains elusive. In *Axin*^*Fu*^ mice, the variable DNA methylation levels of IAP inversely correlate with the severities of the kinky tail phenotype, and penetrant mice tend to produce more penetrant offspring [38]. DNA methylation undergoes two waves of reprogramming during fertilization and germ line specification [63] and IAP seems to be resistant to these reprogramming events [38], which may explain the transgenerational inheritance of the phenotypes in *A*^*vy*^ and *Axin*^*Fu*^ mice. However, in *Axin*^*cL1*^ mice, the kinky tail phenotype occurs as long as the cL1 insertion is present, and the penetrance is fixed at ~ 70–80%. Therefore, the kinky phenotype most likely represent a genetic phenomenon at first glance, and the stable inheritance of this phenotype across multiple generations in *Axin*^*cL1*^ mice appears to be a simple genetic, rather than an epigenetic, transmission, i.e., a cL1 insertional mutation causes the phenotype in each generation. However, partial penetrance (70–80%) of the phenotype can only be explained by an epigenetic mechanism. Our data have linked H3K9ac to the aberrant splicing events, but it remains unknown how such an alteration in histone modifications causes aberrant splicing at a rate of 70–80% rather than 100%.

In summary, we show that insertion of a chimeric L1 into intron 6 of *Axin1* affects histone modification patterns on cL1 and its neighboring regions, leading to the production of an aberrant *Axin1* transcript correlated with the kinky tail phenotype. This mechanism is different from that previously identified in mice with spontaneous IAP retrotransposition (e.g., *Axin*^*Fu*^ and *A*^*vy*^ mice), which results from DNA methylation changes. *Axin1* locus may serve as an ideal genomic location for studying the effects of new retrotransposition events on target gene function in mice in vivo.

## Conclusions

Despite their widespread distribution in the human genome, effects of retrotransposons on their host genes and nearby genome have not been exhaustively investigated in vivo. Here, we show that insertion of a chimeric L1 into intron 6 of *Axin1* locus in mice could induce the kinky tail phenotype due to the production of an aberrantly spliced transcript isoform, which is associated with altered histone modifications rather than DNA methylation changes. Together with previous reports, our data strongly suggest that *Axin1* is an ideal locus for studying the effects of retrotransposition on host gene expression and nearby genome activities in vivo.

## Methods

### Animal use and care

All the mice used in this study were on C57Bl/6 J background, and housed under specific pathogen-free conditions in a temperature- and humidity- controlled animal facility at the University of Nevada, Reno.

### Generation of knock-in mice and breeding scheme

gRNAs were designed using the MIT website (https://zlab.bio/guide-design-resources) and cloned into pX330 plasmid as previously described [42, 43]. The gRNAs were in vitro transcribed using HiScribe™ T7 High Yield RNA Synthesis Kit (E2040S, NEB) and purified using RNA Clean & Concentrator™-5 (R1013, Zymo Research). Cas9 mRNA was purchased from TriLink BioTechnologies (L7606). The IAP LTR and the chimeric L1 were synthesized by IDT, and two homology arms (~ 1 kb) flanking the gRNA cutting sites of *A* or *Axin1* locus were amplified by Q5® Hot Start High-Fidelity 2X Master Mix (M0494S, NEB) from mouse tail genomic DNA. Donor DNA templates that contain homology arms and the IAP LTR or the chimeric L1 were generated with NEBuilder® HiFi DNA Assembly Master Mix (E2621L, NEB). The gRNAs, Cas9 mRNA and donor DNA template were microinjected into mice zygotes of FVB/NJ × C57BL/6 J background for *Axin1* locus knock-in and C57BL/6 J for *A* locus knock-in. The genomic DNA of founder mice from tail tips or ear snips were extracted for PCR-based genotyping. Founder mice were outcrossed with C57BL/6 J WT to obtain heterozygous F1 *(Axin*^*+/IAP*^ or *Axin*^*+/cL1*^). For *A*^*IAP*^, F1 s were outcrossed with C57BL/6 J WT to obtain heterozygous F2 s, and coat color was recorded. For *Axin*^*IA*P^, F1 s were outcrossed with C57BL/6 J WT to obtain heterozygous F2 s, and tail phenotype was examined. For *Axin*^*cL1*^, F1 s were outcrossed with C57BL/6 J WT to obtain heterozygous F2 s, and tail phenotype was recorded. Penetrant and silent heterozygous F2 s, F3 s, and F4 s were further outcrossed with C57BL/6 J WT to obtain the breeding data across multiple generations. Primers used for all the constructs are listed in Additional file 4: Table S3.

### Mouse genotyping

Mouse tail or ear snip samples were lysed in a lysis buffer containing 40 mM NaOH (221465, Sigma Aldrich) and 0.2 mM EDTA (46–034-CI, Corning) for 1 h at 95 °C, followed by neutralization with the same volume of the neutralizing buffer containing 40 mM Tris-HCl (15567027, Thermo Fisher Scientific). PCR-based genotyping of the *Axin*^*cL1*^, *Axin*^*IAP*^ and *A*^*IAP*^ was conducted using the GoTaq Green master mix (M7123, Promega) or Platinum™ SuperFi™ Green PCR Master Mix (12359010, Thermo Fisher Scientific). Primers used for genotyping are listed in Additional file 4: Table S3.

### Dual luciferase assay

Different fragments of the repetitive sequences were amplified from donor templates and then used to replace the SV40 early promotor that drives the expression of the *Renilla* luciferase-coding sequence in the psiCHECK-2 plasmid (C8021, Promega). HEK293 cells were transfected with psiCHECK-2 containing the different fragments from the repetitive sequences using Lipofectamine 2000 (11668019, Thermo Fisher Scientific) in a 24-well cell culture plate (CLS3527-100EA, Corning). After 24 h, cells were lysed and used for the Dual Luciferase Assay (E1910, Promega) according to the manufacturer’s instructions. The psiCHECK-2 and psiCHECK-2 vectors with deletion of the SV40 early promotor of the *Renilla* luciferase-coding sequence were used as positive and negative controls, respectively. *Renilla* luciferase signals were normalized to *Firefly* luciferase signals to correct the transfection efficiency. Primers used for all the constructs are listed in Additional file 4: Table S3.

### DNA, RNA extraction and cDNA synthesis

DNA and RNA were extracted from kidneys and tail snips from penetrant and silent mice using the Quick-DNA Plus Kits (D4074, Zymo Research) and mirVana miRNA Isolation Kit (AM1560, Thermo Fisher Scientific), respectively, according to the manufacturer’s instructions. Briefly, kidney or tail samples were homogenized in 600 μL of Lysis/Binding Buffer with homogenizer (D1000, Benchmark), followed by centrifugation to remove cell debris. The supernatant was passed through a column, in which the genomic DNA was retained, whereas the RNA got eluted. For genomic DNA extraction, the column containing genomic DNA was treated with a genomic lysis buffer at room temperature for 10 min, followed by washing with a DNA Pre-Wash Buffer once and a g-DNA Wash Buffer twice. The genomic DNA was eluted with nuclease-free water and stored at − 80 °C for further use. For RNA extraction, 60 μL of miRNA Homogenate Additive was added into the flow-through followed by incubation on ice for 10 min. The mixture was subjected to Phenol: Chloroform RNA extraction, and total RNA was isolated according to the manufacturer’s instructions. cDNA synthesis was performed using SuperScript II Reverse Transcriptase (18064014, Thermo Fisher Scientific) with random primers. qPCR and long-range PCR were performed using the Fast SYBR Green Master Mix (4385612, Thermo Fisher Scientific) and PrimeSTAR GXL DNA Polymerase (R050B, TaKaRa), respectively. Primers used for qPCR and long-range PCR are listed in Additional file 4: Table S3.

### Bisulfite sequencing

Genomic DNA samples were bisulfite-converted using the EZ DNA Methylation-Gold™ Kit (D5005, Zymo Research). PCR was performed using the TaKaRa EpiTaq™ HS enzyme (for bisulfite-treated DNA) (R110B, TaKaRa), which is more tolerant to dUTP-containing templates, with Tm at 55 °C for 40 cycles. PCR products were ligated into the pGEM®-T Easy Vector (A1360, Promega) for Sanger sequencing. Primers used for bisulfite sequencing are listed in Additional file 4: Table S3.

### Methylated DNA immunoprecipitation-qPCR (MeDIP-qPCR)

MeDIP was performed using a Methylated DNA immunoprecipitation kit (ab117133, Abcam) according to instructions of the manufacturer. In brief, 100 μL of the antibody buffer and 1 μL anti-5-methylcytosine or mouse IgG antibody were added into strip wells and incubated at room temperature for 1 h. During the incubation, tail genomic DNA was sheared by a focused-ultrasonicator (M220, Covaris) in the reaction buffer. The sheared DNA ranged between 200 and 1000 bp in size and was denatured at 95 °C for 2 min followed by incubation on ice. An aliquot of 5 μL of the denatured DNA was used as input DNA. The strip wells bound with antibody were washed with 150 μL of the antibody buffer once and 150 μL of the wash buffer once, followed by incubation with the sheared DNA at room temperature for 2 h. Then the strip wells were washed with the wash buffer three times. The antibody-enriched DNA was eluted with a DNA release buffer containing proteinase K and purified with columns. qPCR was performed to identify DNA methylation levels. Primers used for MeDIP-qPCR are listed in Additional file 4: Table S3.

### Northern blot

Northern blot analyses were performed using a NorthernMax® Kit (AM1940, Thermo Fisher Scientific) and a Biotin Chromogenic Detection Kit (K0662, Thermo Fisher Scientific) following the manufacturer’s instructions. Briefly, RNA extracted from kidney was mixed with 3 volumes of a formaldehyde-containing loading dye followed by denaturation at 65 °C for 15 min. The denatured RNA was the fractionated through 1 × MOPS Gel Running Buffer (1% denaturing gel with a voltage of 140 V for 30 min). Then the RNA was transferred onto a BrightStar®-Plus Positively Charged Nylon Membrane (AM10100, Thermo Fisher Scientific) using Novex™ Semi-Dry Blotter (SD1000, Thermo Fisher Scientific) in 1× TBE buffer with a voltage of 20 V for 30 min. After transfer, the membrane was rinsed 1× Gel Running Buffer, then crosslinked in Spectrolinker™ XL-1500 UV crosslinker (Spectronics Corporation) followed by baking at 80 °C for 15 min. The crosslinked membrane was prehybridized at 65 °C in a preheated ULTRAhyb Buffer in a roller bottle in a hybridization oven at 42 °C for 30 min, followed by incubation with 10pM biotinylated probe (IDT) in the ULTRAhyb Buffer at 42 °C overnight. After rinsing with 1 × Blocking/Washing Buffer for 5 min three times at room temperature, the membrane was blocked with 1 × Blocking Buffer for 30 min in a shaker at room temperature. Following blocking, the membrane was incubated with Streptavidin-AP conjugate for 1 h at room temperature, then washed with 1 × Blocking/Washing Buffer for 5 min three times and 1× detection buffer for 10 min. Then the membrane was incubated with freshly prepared NBT/BCIP Substrate Solution at room temperature in the dark. 2 h later, the reaction was stopped by rinsing with double deionized water. Probes used for Northern blot are listed in Additional file 4: Table S3.

### Chromatin immunoprecipitation followed by quantitative PCR (ChIP-qPCR)

ChIP-qPCR was performed as previously described [64]. Briefly, tail snips were lysed on ice for 30 min in 600 μl of buffer 1 plus detergents [15 mM Tris-HCl (pH 7.5) (15567027, Thermo Fisher Scientific), 60 mM KCl (P217–500, Fisher Scientific), 5 mM MgCl_2_ (BP214–500, Fisher Scientific) and 0.1 mM EGTA (O2783–100, Fisher Scientific), 0.3 M sucrose (freshly added) (0335-5KG, Amresco), 10 mM DTT (freshly added) (GE17–1318-02, GE Healthcare), 0.25% (volume/volume) NP-40 (NP40S-500ML, Sigma Aldrich) and 0.5% (weight/volume) sodium deoxycholate (freshly prepared) (D6750-100G, Sigma Aldrich)]. Then 600 μl of MNase buffer [85 mM Tris-HCl, pH 7.5, 3 mM MgCl_2_, 2 mM CaCl2 (C7902-500G, Sigma Aldrich) and 0.3 M sucrose (freshly added)] was added into the lysed solution. The mixture was aliquoted into 200 μl per tube to obtain sufficient digestion, followed by Micrococcal Nuclease (M0247S, NEB) digestion at 37 °C in a thermomixer for 5 min and then terminated by adding 2ul of 0.5 M EDTA (46–034-CI, Corning) and incubation on ice for 5 min. The digested sample was then centrifuged at 15,000×g for 10 min at room temperature to remove cell debris, followed by adding protease inhibitors to the chromatin. 200 μl of the mixture was saved as input DNA. After preclearing of the chromatin with blocked protein G beads (10004D, Thermo Fisher Scientific), 3 μl of H3K9ac antibody (ab4441, Abcam) was added into the precleared chromatin and incubated at 4 °C overnight. Then the chromatin was incubated with blocked protein G beads at 4 °C for 4 h, followed by washing with wash buffer A (50 mM Tris-HCl (pH 7.5), 10 mM EDTA and 75 mM NaCl (BP358–10, Fisher Scientific)) once and wash buffer B (50 mM Tris-HCl (pH 7.5), 10 mM EDTA and 125 mM NaCl) twice. Then the chromatin was eluted by resuspending in 150 μl of elution buffer (1% (weight/volume) SDS (L4509-500G, Sigma Aldrich) in TE) at 25 °C in a thermomixer twice. The eluted chromatin was then subjected to RNase A (EN0531, Thermo Fisher Scientific) and proteinase K (P8107S, NEB) digestion followed by phenol/chloroform extraction of DNA. The pull-down DNA and input DNA were used for qPCR using the Fast SYBR Green Master Mix (4385612, Thermo Fisher Scientific). Primers used for ChIP-qPCR are listed in Additional file 4: Table S3.

### Statistical analysis

All data were presented as mean ± SEM, and statistical differences were assessed by the Two-sample t test unless stated otherwise. *p* < 0.05 was considered as significant differences.

## Additional files

Additional file 1: **Figure S1, S2, S3 and Supplemental notes.** Generation of *Axin*^*IAP*^ and *A*^*IAP*^ founder mice and copy number variation assays for *Axin*^*cL1*^ mice (Figure S1); promoter activity analyses (Figure S2); DNA methylation levels around cL1 in penetrant and silent *Axin*^*cL1*^ mice (Figure S3); and annotated sequences of retrotransposons used in this study (Supplemental notes). (PDF 5204 kb)

Additional file 2: **Table S1.** Orf2 BLAT results. (XLSX 52 kb)

Additional file 3: **Table S2.** MaLR BLAT results. (XLSX 48 kb)

Additional file 4: **Table S3.** Oligos used in this study. (XLSX 11 kb)

## Abbreviations

5mC: 5-methylcytosine
*A*^*vy*^: Agouti viable yellow
*Axin*^*cL1*^: *Axin1* ^*+/cL1*^
*Axin*^*Fu*^: *Axin* ^*Fused*^
*Axin*^*Kb*^: *Axin* ^*Knobby*^
*Axin*^*Ki*^: *Axin* ^*Kinky*^
BLAT: BLAST-like alignment tool
Cas9: CRISPR associated protein 9
ChIP: Chromatin immunoprecipitation
cL1: Chimeric L1
CRIPSR: Clustered regularly interspaced short palindromic repeats
ERV: Endogenous retroviruse
IAP: Intracisternal A-type particle
L1: LINE-1
LINE: Long interspersed element
LTR: Long terminal repeat
MaLR: Mammalian apparent LTR retrotransposon
MeDIP: Methylated DNA immunoprecipitation
ORF: Open reading frame
qPCR: Quantitative PCR
RE: Restriction enzyme
SINE: Short interspersed elements
SVA: SINE-VNTR-Alu
TE: Transposable element
TSDs: Target site duplications
UTR: Untranslated region
WT: Wild type
