# Supplementary material for: Insertion of a chimeric retrotransposon sequence in mouse Axin1 locus causes metastable kinky tail phenotype
Source: Mob DNA. 2019 May 3;10:17. doi: 10.1186/s13100-019-0162-7 (PMC6500023; doi:10.1186/s13100-019-0162-7)

Additional file1

Insertion of a chimeric retrotransposon sequence in mouse *Axin1* locus causes metastable kinky tail phenotype

Zhuqing Wang[1], Hayden McSwiggin[1], Simon J. Newkirk[3], Yue Wang[1], Daniel Oliver[1], Chong Tang[1], Sandy Lee[1], Shawn Wang[1], Shuiqiao Yuan[1], Huili Zheng[1], Ping Ye[2,3], Wenfeng An[3] and Wei Yan[1,4,5,]*

[1]Department of Physiology and Cell Biology, University of Nevada, Reno School of Medicine, Reno, NV 89557; [2]Avera McKennan Hospital and University Health Center, Sioux Falls, SD 57108; [3]Department of Pharmaceutical Sciences, South Dakota State University, Brookings, SD 57007; [4]Department of Obstetrics and Gynecology, University of Nevada, Reno School of Medicine, Reno, NV 89557; and [5]Department of Biology, University of Nevada, Reno, Reno, NV 89557

This file contains three figures (Figs. S1-S3) and one supplemental note with annotated sequences of retrotransposons used in this study.

Fig. S1

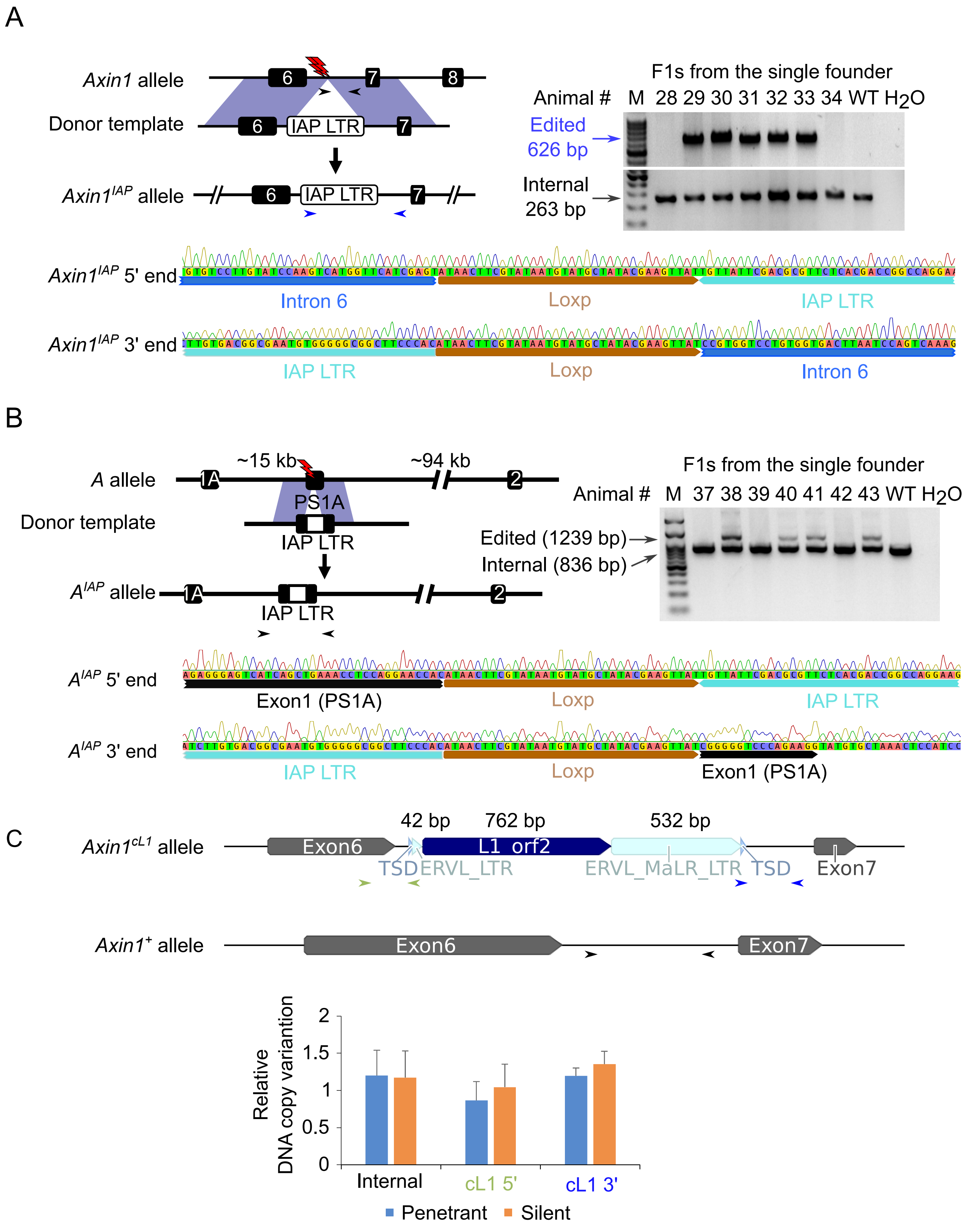

A
Axin1 allele
Donor template
6
7
8
IAP LTR
Axin1^IAP allele
F1s from the single founder
Animal # M 28 29 30 31 32 33 34 WT H2O
Edited 626 bp
Internal 263 bp
Axin1^IAP 5' end
Intron 6
Loxp
IAP LTR
Axin1^IAP 3' end
IAP LTR
Loxp
Intron 6
B
A allele
~15 kb
~94 kb
1A
2
PS1A
Donor template
IAP LTR
A^IAP allele
IAP LTR
F1s from the single founder
Animal # M 37 38 39 40 41 42 43 WT H2O
Edited (1239 bp)
Internal (836 bp)
A^IAP 5' end
Exon1 (PS1A)
Loxp
IAP LTR
A^IAP 3' end
IAP LTR
Loxp
Exon1 (PS1A)
C
Axin1^cL1 allele
42 bp
762 bp
532 bp
Exon6
L1_orf2
TSD
ERVL_LTR
ERVL_MaLR_LTR
TSD
Exon7
Axin1^+ allele
Exon6
Exon7
Relative DNA copy varaintion
2
1.5
1
0.5
0
Internal
cL1 5'
cL1 3'
Penetrant
Silent

Fig. S1 Generation of *Axin*^{IAP} and *A*^{IAP} founder mice and copy number variation assays for *Axin*^{cL1} mice. **a** The strategy used to generate *Axin*^{IAP} founder mice using CRISPR/Cas9 (upper left panel) and representative genotyping results showing detection of the *Axin1*^{IAP} insertion (upper right panel), as well as validation by Sanger sequencing (lower panel). In the upper left panel, the red lightning bolts represent the gRNAs used to target the reverse strands of the genomic DNA; the black arrows show the position of internal primers in the *Axin1*$^{+}$ allele, whereas the blue ones indicate that of IAP primers in the *Axin1*^{IAP} allele. The expected size of PCR products in the upper left panel is indicated in the same color as in the upper right panel. **b** Strategy used to generate *A*^{IAP} founder mice using CRISPR/Cas9 (upper left panel) and representative genotyping results showing detection of the *A*^{IAP} insertion (right upper panel), as well as validation by Sanger sequencing (lower panel). The red lightning bolts represent the gRNAs used to target the reverse strand of the genomic DNA. The black arrows show the position of external primers in the *A*$^{+}$ and *A*^{IAP} alleles. The expected size of PCR products in the upper left panel is indicated in the upper right panel. **c** qPCR-based copy number variation assays on penetrant and silent *Axin*^{cL1} (*Axin1*$^{+/cL1}$) mice. Data are presented as means ± SEM, n=3. As marked in upper panels, primers used for detection of cL1 5'end were in light green, whereas primers for analyzing cL1 3' end were in blue. Internal primers in black were used for amplifying an internal fragment as controls.

Fig. S2

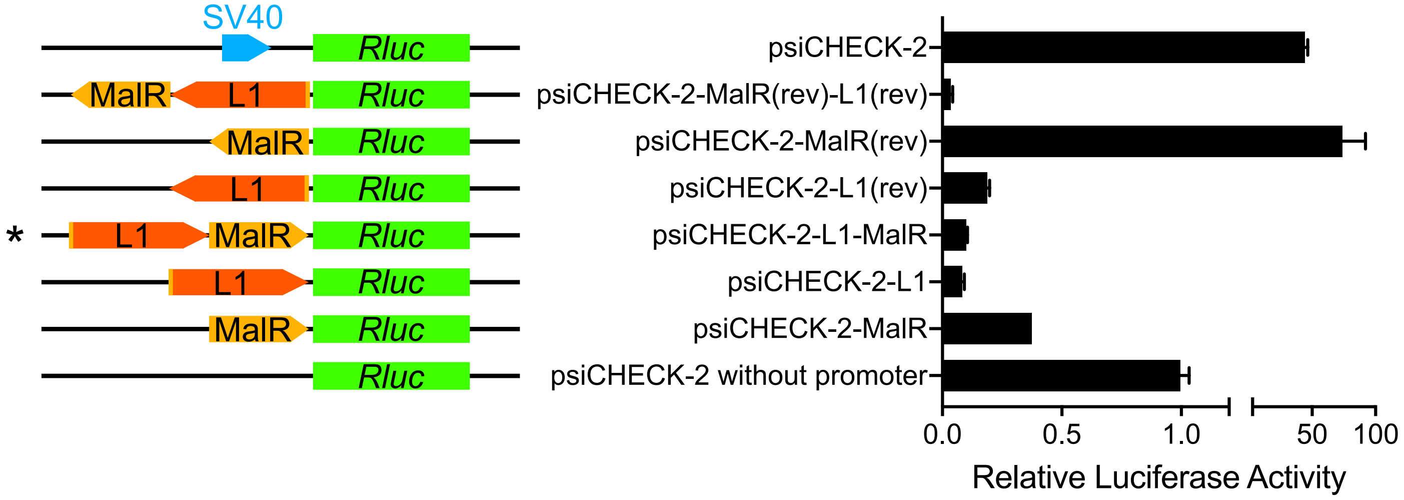
SV40
Rluc
MalR
L1
*
psiCHECK-2
psiCHECK-2-MalR(rev)-L1(rev)
psiCHECK-2-MalR(rev)
psiCHECK-2-L1(rev)
psiCHECK-2-L1-MalR
psiCHECK-2-L1
psiCHECK-2-MalR
psiCHECK-2 without promoter
0.0
0.5
1.0
50
100
Relative Luciferase Activity

Fig. S2 Promoter activity analyses. Dual luciferase reporter-based promoter activity assays for various parts of the chimeric L1 (asterisk) used in this study. SV40 early promoter was used as a positive control, and *Rluc* without promoter was used as a negative control. *Rluc* luciferase activity was normalized to *Firefly* luciferase activity, which is located in the same plasmid as *Rluc* luciferase.

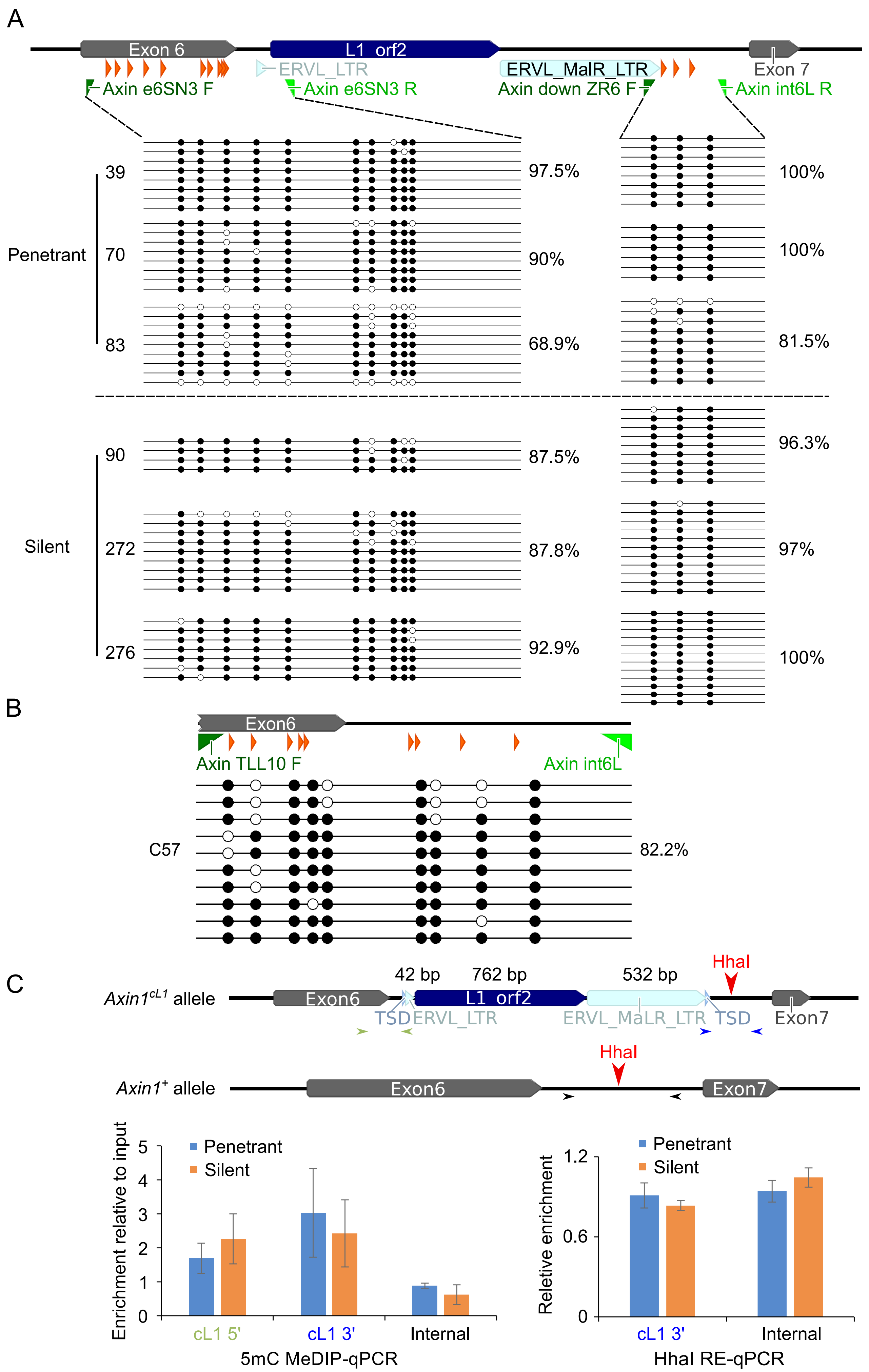

A
Exon 6
L1 orf2
ERVL_LTR
ERVL_MalR_LTR
Exon 7
Axin e6SN3 F
Axin e6SN3 R
Axin down ZR6 F
Axin int6L R
Penetrant
39
70
83
97.5%
90%
68.9%
100%
100%
81.5%
Silent
90
272
276
87.5%
87.8%
92.9%
96.3%
97%
100%
B
Exon6
Axin TLL10 F
Axin int6L
C57
82.2%
C
Axin1cL1 allele
42 bp
762 bp
532 bp
HhaI
Exon6
L1 orf2
TSD
ERVL_LTR
ERVL_MaLR_LTR
TSD
Exon7
Axin1+ allele
HhaI
Exon6
Exon7
Enrichment relative to input
Penetrant
Silent
0
1
2
3
4
5
cL1 5'
cL1 3'
Internal
5mC MeDIP-qPCR
Reletive enrichment
Penetrant
Silent
0
0.6
1.2
cL1 3'
Internal
HhaI RE-qPCR

Fig. S3. DNA methylation levels around cL1 in penetrant and silent *Axin*^{cL1} mice. a Bisulfite sequencing of two regions flanking cL1 in penetrate and silent *Axin*^{cL1} mice. The blacked circle indicates methylated cytosine (5mC), whereas the open circle marks unmethylated cytosine in the CG site. The number of penetrant and silent mice used is marked to the left, whereas the percentage of 5mC detected is shown to the right. Orange arrowheads represent the CG sites, and positions of forward and reverse primers used are shown in dark and light green, respectively. **b** Bisulfite sequencing of the same two regions in wild-type (WT C57Bl6/J) mice. The percentage of 5mC detected is shown to the right. Orange arrowheads represent the CG sites, and positions of forward and reverse primers used are shown in dark and light green, respectively. **c** MeDIP-qPCR (lower left panel) and HhaI Restriction enzyme (RE)-qPCR (lower right panel) analyses of two regions flanking cL1 in penetrate and silent *Axin*^{cL1} mice. MeDIP-qPCR data were normalized to input DNA. Data are presented as means ± SEM, n=3. RE-qPCR data were normalized to *Axin*^{cL1} 5' end, which does not contain HhaI restriction site. Data are presented as means ± SEM, n=3. Primers used for MeDIP-qPCR (lower left panel) and RE-qPCR (lower right panel) are indicated in the upper panel with corresponding colors.

Supplemental notes

A^{IAP}

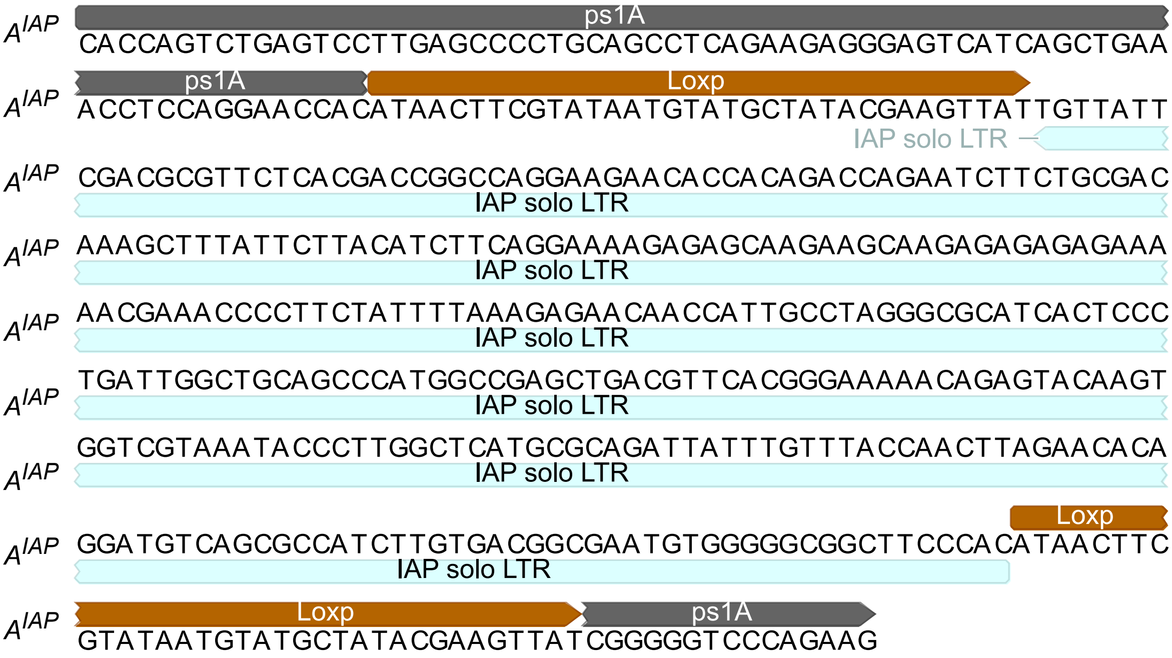

Axin^{IAP}

Exon6
Axin^{IAP} GAGGAAGAAGGGGAGGATGGTGAAATGCCTTCTGGCCCCATGGCAAGTCACAAGC

Exon6
Axin^{IAP} TGCCTTCTGTCCCAGCTTGGCACCATTTCCCACCCCGCTATGTGGATATGGGCTG

Exon6
Axin^{IAP} CTCTGGACTGCGGGATGCCCATGAGGAGAATCCTGAGAGCATCCTGGATGAGCAC

Exon6
Axin^{IAP} GTGCAAAGGGTCATGAGGACACCTGGCTGCCAGTCACCTGGCCCAGGCCACCGCT

Exon6
Axin^{IAP} CTCCTGACAGTGGGCATGTGGCTAAGACTGCAGTGCTAGGGGGTACAGCCTCCGG

Exon6
Axin^{IAP} GCATGGGAAGCATGTTCCTAAGTTAGGGTTGAAGCTGGATACAGCTGGCCTGCAC

Exon6
Axin^{IAP} CATCATAGACATGTCCACCACCATGTTCACCATAATTCAGCTAGACCTAAGGAGC

Exon6
Axin^{IAP} AAATGGAGGCTGAAGTTGCCCGCAGGGTCCAGAGCAGCTTCTCGTGGGGCCCAGA

Exon6
Axin^{IAP} AACACATGGTCATGCCAAGCCCCGGAGCTATTCCGAGAACGCAGGCACCACCCTC

Exon6
Axin^{IAP} AGTGCTGGGGATTTGGCCTTTGGGTGAGTCTTGATCCAGCCTTCTTAATGTTTCC

loxp
Axin^{IAP} AGTGTGTCCTTGTATCCAAGTCATGGTTCATCGAGTATAACTTCGTATAATGTAT

loxp
Axin^{IAP} GCTATACGAAGTTATTGTTATTCGACGCGTTCTCACGACCGGCCAGGAAGAACAC
IAP solo LTR

Axin^{IAP} CACAGACCAGAATCTTCTGCGACAAAGCTTTATTCTTACATCTTCAGGAAAAGAG
IAP solo LTR

Axin^{IAP} AGCAAGAAGCAAGAGAGAGAGAAAAACGAAACCCCTTCTATTTTAAAGAGAACAA
IAP solo LTR

Axin^{IAP} CCATTGCCTAGGGCGCATCACTCCCTGATTGGCTGCAGCCCATGGCCGAGCTGAC
IAP solo LTR

Axin^{IAP} GTTCACGGGAAAAACAGAGTACAAGTGGTCGTAAATACCCTTGGCTCATGCGCAG
IAP solo LTR

Axin^{IAP} ATTATTTGTTTACCAACTTAGAACACAGGATGTCAGCGCCATCTTGTGACGGCGA
IAP solo LTR

Loxp
Axin^{IAP} ATGTGGGGGCGGCTTCCCACATAACTTCGTATAATGTATGCTATACGAAGTTATC
IAP solo LTR

Axin^{IAP}

Intron 6
Axin^{IAP} CGTGGTCCTGTGGTGACTTAATCCAGTCAAAGTCAGACAATACCCGGGCCAGGGG

Intron 6
Axin^{IAP} CCAGTCAGACTCCAGGCCTTGCTATGACTGGATGAGGAGGGGCGCACTTTGGGTT

Intron 6
Axin^{IAP} TTTCTTGGGCTCTGGTCAGCATGGCTCTCCTACCATAGTAAAACAGACCACAGAC

Intron 6
Axin^{IAP} AGCTGACCTTCACAGAGGCCTCAGCCTTCACAGTGGGTTGAAGGACTTCTTGTTG

Intron 6
Axin^{IAP} AGCTTGAGGGTTCAGGAAGTAGCAAGAAAGACCAGAGTGACCTCCTATGTCTTGT

Intron 6 Exon7
Axin^{IAP} TCTCCCAGTGGTAAAACTAGTGCACCTTCCAAAAGAAACACCAAGAAGGCTGAAT

Exon7
Axin^{IAP} CTGGGAAGAATGCCAATGCTGAGGTACCCAGTACCACAGAGGACGCTGAGAAGAA

Exon7
Axin^{IAP} CCAGAAGATCATGCAGTGGATCATTGAGGGAGAGAAGGAGATCAGTAGACACCGG

Exon7
Axin^{IAP} AAGGCAGGCCATGG

$Axin^{cL1}$

Exon6
$Axin^{cL1}$ GAGGAAGAAGGGGAGGATGGTGAAATGCCTTCTGGCCCCATGGCAAGTCACAAGC

Exon6
$Axin^{cL1}$ TGCCTTCTGTCCCAGCTTGGCACCATTTCCCACCCCGCTATGTGGATATGGGCTG

Exon6
$Axin^{cL1}$ CTCTGGACTGCGGGATGCCCATGAGGAGAATCCTGAGAGCATCCTGGATGAGCAC

Exon6
$Axin^{cL1}$ GTGCAAAGGGTCATGAGGACACCTGGCTGCCAGTCACCTGGCCCAGGCCACCGCT

Exon6
$Axin^{cL1}$ CTCCTGACAGTGGGCATGTGGCTAAGACTGCAGTGCTAGGGGGTACAGCCTCCGG

Exon6
$Axin^{cL1}$ GCATGGGAAGCATGTTCCTAAGTTAGGGTTGAAGCTGGATACAGCTGGCCTGCAC

Exon6
$Axin^{cL1}$ CATCATAGACATGTCCACCACCATGTTCACCATAATTCAGCTAGACCTAAGGAGC

Exon6
$Axin^{cL1}$ AAATGGAGGCTGAAGTTGCCCGCAGGGTCCAGAGCAGCTTCTCGTGGGGCCCAGA

Exon6
$Axin^{cL1}$ AACACATGGTCATGCCAAGCCCCGGAGCTATTCCGAGAACGCAGGCACCACCCTC

Exon6 | Intron 6
$Axin^{cL1}$ AGTGCTGGGGATTTGGCCTTTGGGTGAGTCTTGATCCAGCCTTCTTAATGTTTCC

TSD
Intron 6 | 5' extra nucleotides
$Axin^{cL1}$ AGTGTGTCCTTGTATCCAAGTCATGGTTCATCTATCTGTAAGTTCTGTGACTCTA

5' extra nucleotides | L1_orf2
$Axin^{cL1}$ GAGAACCCTGACTAATACACCAAGGAAATTTAAAAAAAAATCATCAGATTCTACT

L1_orf2
$Axin^{cL1}$ ACAAAAGCTTATACTAAACAAAACTGGAAAACCTGAATAAATGGATGATTTTCTA

L1_orf2
$Axin^{cL1}$ GACAGATACGATGTACCAAAGTTAAATCCAGATCAGATAAACCATCTAAACAGTC

L1_orf2
$Axin^{cL1}$ CCATAACCAGAAGCATTCACTAAAAGTCTCCTAACCAAATTAATTCCAGGGCCAG

L1_orf2
$Axin^{cL1}$ ATGCTCTCAATGCAGAATTCTATAAGACTTTCAAACAAAACCTAAAACCAATACT

L1_orf2
$Axin^{cL1}$ TTCCAAACTATTCCACAATATAAAAGTAGAAGGAACAACTACCTAATTCATTCTA

L1_orf2
$Axin^{cL1}$ TGAACCCACAGTTATGCTGAAACCTCAACGACACAAAGACCCAACAAAGAAAGAG

L1_orf2
$Axin^{cL1}$ AACTTCAGACCAATTTTGTTTACAGTATCTGTGCAAAAATACTCAATGAAATTCT

L1_orf2
$Axin^{cL1}$ AGCAAACTGAATCCAATAATATACCAAAATGATCACTCACCAGGGTCAAGTAGGC

L1_orf2
$Axin^{cL1}$ TTAATCTCAGGGATGCAGGGATGGTTCAATATACAGAAATCCATCAATATAATCT

L1_orf2
$Axin^{cL1}$ ATTATATGAACAAACTCAAAGACAAAAAACCGACATGATCATCTCATTAGACGCT

L1_orf2
$Axin^{cL1}$ GAAAAAGCCTTTGATAAAATACAACTCTCCTTCATGTTAAAAGACTTGGAAATAT

L1_orf2
$Axin^{cL1}$ GAGGAATTCGTGTAACTTACCTAAACATAATAAAAGCAATATACAGCAAACCAAT

L1_orf2
$Axin^{cL1}$ AGCCAACATCAAATTAAATGGAGAGAAACTTGAAGCAATCTCACTGAAATCAGAG

MaLR_LTR
L1_orf2
$Axin^{cL1}$ CTAAGACAAGGTTGTAATGTGTAAGAGTCACCCAGGTGGTACTGGTTTTGAAGGC

MaLR_LTR
$Axin^{cL1}$ ATGAAGGAGTTGAGCAGAACAGCTGAGGCTTGGCACTGTGAGAGGCCATGGAAGG

MaLR_LTR
$Axin^{cL1}$ CCATTGGTGAAAGTCCAGCCTCATTTGCAATTGATGGCCCAGGACTGAAGGGGTC

MaLR_LTR
$Axin^{cL1}$ ATGCAGTGTTTTGGAGATGCCAGTACCATGAGATGACCACCAAGAACAGCAGCAG

MaLR_LTR
$Axin^{cL1}$ CAGTGGAGTACAGGCATCTGGAGCCTAGAGGATGACGCGTGTGCTACAAAGGGCC

Axin^{cL1} (continued)

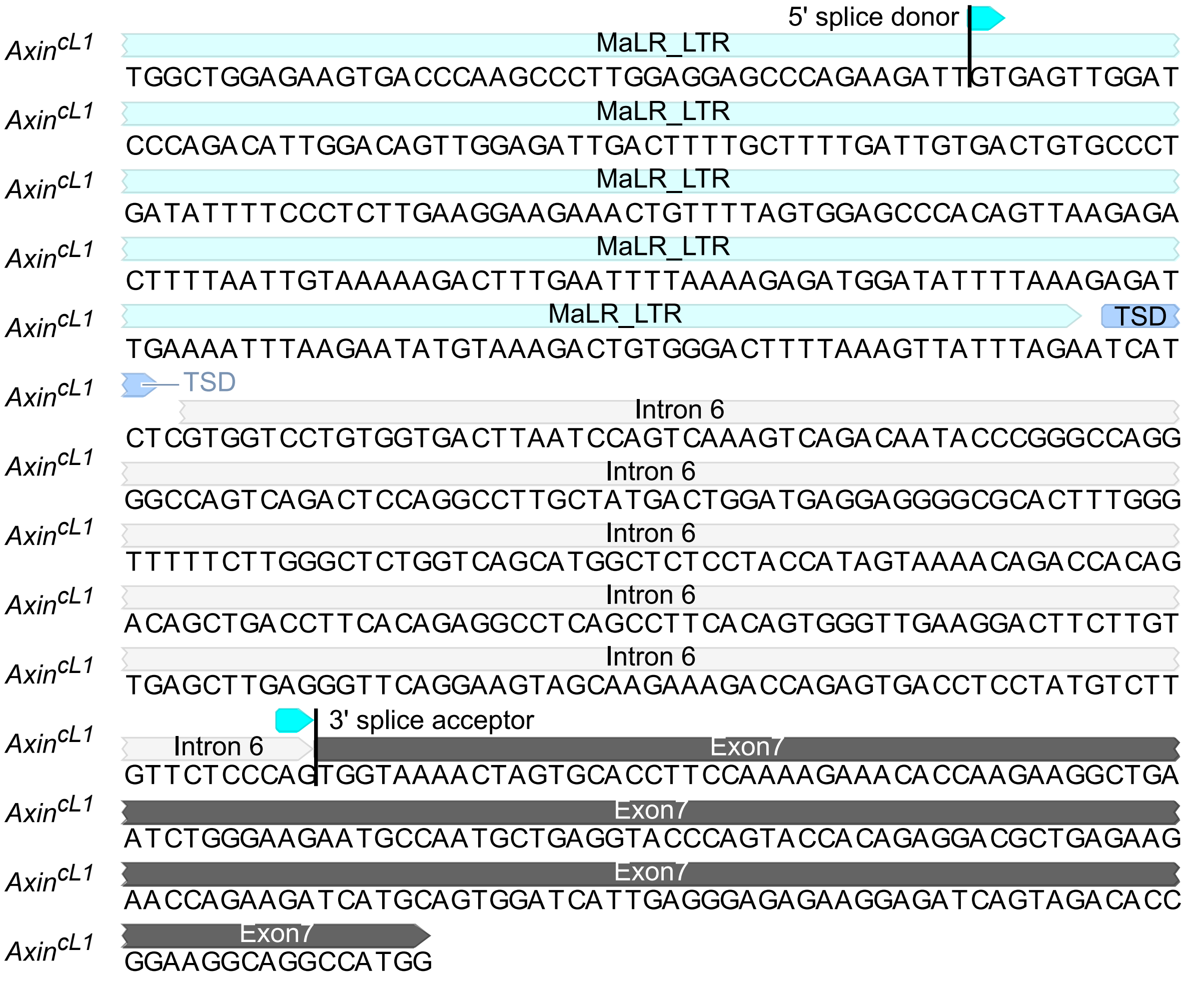

Supplement: Supplementary file 1 — Figure S1, S2, S3 and Supplemental notes. Generation of AxinIAP and AIAP founder mice and copy number variation assays for AxincL1 mice (Figure S1); promoter activity analyses (Figure S2); DNA methylation levels around cL1 in penetrant and silent AxincL1 mice (Figure S3); and annotated sequences of retrotransposons used in this study (Supplemental notes). (PDF 5204 kb) [file 13100_2019_162_MOESM1_ESM.pdf]
